# Evaluation of carbonic anhydrase and paraoxonase inhibition activities and molecular docking studies of highly water-soluble sulfonated phthalocyanines

**DOI:** 10.3906/kim-2007-21

**Published:** 2020-12-16

**Authors:** Emre GÜZEL, Fatih SÖNMEZ, Sultan ERKAN, Kübra ÇIKRIKÇI, Adem ERGÜN, Nahit GENÇER, Oktay ARSLAN, Makbule B KOÇAK

**Affiliations:** 1 Department of Fundamental Sciences, Faculty of Technology, Sakarya University of Applied Sciences, Sakarya Turkey; 2 Pamukova Vocational School, Sakarya University of Applied Sciences, Sakarya Turkey; 3 Chemistry and Chemical Processing Technologies, Yıldızeli Vocational School, Sivas Cumhuriyet University, Sivas Turkey; 4 Department of Chemistry, Faculty of Arts and Science, Balıkesir University, Balıkesir Turkey; 5 Department of Chemistry, Faculty of Arts and Science, İstanbul Technical University, İstanbul Turkey

**Keywords:** Phthalocyanine, sulfonated, water-soluble, paraoxonase, carbonic anhydrase, enzyme inhibition, molecular docking

## Abstract

The investigation of carbonic anhydrase and paraoxonase enzyme inhibition properties of water-soluble zinc and gallium phthalocyanine complexes ( **1** and **2** ) are reported for the first time. The binding of p-sulfonylphenoxy moieties to the phthalocyanine structure favors excellent solubilities in water, as well as providing an inhibition effect on carbonic anhydrase (CA) I and II isoenzymes and paraoxonase (PON1) enzyme. According to biological activity results, both complexes inhibited hCA I, hCA II, and PON1. Whereas **1** and **2** showed moderate hCA I and hCA II (off-target cytosolic isoforms) inhibitory activity (Ki values of 26.09 µM and 43.11 µM for hCA I and 30.95 µM and 33.19 µM for hCA II, respectively), they exhibited strong PON1 (associated with high-density lipoprotein [HDL]) inhibitory activity (Ki values of 0.37 µM and 0.27 µM, respectively). The inhibition kinetics were analyzed by Lineweaver–Burk double reciprocal plots. It revealed that **1** and **2** were noncompetitive inhibitors against PON1, hCA I, and hCA II. These complexes can be more advantageous than other synthetic CA and PON inhibitors due to their water solubility. Docking studies were carried out to examine the interactions between hCA I, hCA II, and PON1 inhibitors and metal complexes at a molecular level and to predict binding energies.

## 1. Introduction

Phthalocyanines (Pcs), an important family of porphyrinoid complexes, have many applications such as gas sensors [1], solar cells [2], liquid crystals phases [3,4], electrochromic materials [5], and photosensitizer [6–9] in photodynamic therapy (PDT). For these applications, the photophysical and photochemical features of phthalocyanines can be fine-tuned by the introduction of various substituent groups. It is also known that the physical and chemical properties of the complex depend significantly on the nature of the metal atom coordinated to the phthalocyanine ring [8,10,11]. The lack of water solubility of phthalocyanines limits their use in many areas. Also, water solubility is very important for cancer treatments because the complexes are injected into the patient’s bloodstream with a hydrophilic system [12]. Water-soluble phthalocyanines continue to attract attention to their interactions with DNA and their ability to trigger DNA photodamage by accumulating in many cancer cells. Sulfonate, carboxylate, and phosphorus groups can be used from anionic substituents to the macrocyclic ring by various intermediates or directly attached to the phthalocyanine complexes. In particular, the binding of sulfonic acid groups to the phthalocyanine ring has two important effects: they increase the effectiveness of their antitumor properties [13], and by inducing the repulsion of phthalocyanine rings, they become water-soluble as monomers [9,12,14]. Also, in the literature, gallium and zinc metal complexes of the phthalocyanines are examined due to their superior antitumor properties [13]. Thus, these phthalocyanines are beneficial to PDT and various biological applications.

Carbonic anhydrase (CA) isoenzymes are metalloenzymes that catalyze a very easy reaction: the hydration of CO2 to bicarbonate and H+ [15–17]. This key reaction plays a significant role in more pathological and physiological mechanisms associated with ion transport, pH control, and fluid secretion [18]. The inhibition of these isoenzymes is the main goal correlated with the treatment of diverse diseases such as obesity, glaucoma, and epilepsy. More recently, CA inhibition was validated as a novel approach to fighting metastases and tumors. Paraoxonase, which has an important role in living metabolism, is a calcium-dependent enzyme and is also an organophosphate hydrolyzer. These enzymes can hydrolyze aromatic carboxyl esters such as phenylacetate and various lactones. The name “PON” is derived from paraoxon, a common in vitro substrate. Paraoxonase 1 (PON1), which is associated with high-density lipoprotein (HDL) with 355 amino acids, is the most studied member of the mammalian enzyme family. PON1 is considered an important enzyme for two main reasons in metabolism: (i) it protects the system against the neurotoxicity of organophosphates and (ii) oxidizing lipid levels interfere with the onset of atherosclerosis, thus preventing oxidation of low-density lipoproteins, so the activity of PON 1 is considered a risk for atherosclerosis.

Taking these properties into consideration, we focused on water-soluble complexes as a strategy to explain Pcs in conjunction with the sulfonic acid group to study enzyme inhibition, chemical interactions, and theoretical properties. In this regard, water-soluble sulfonated zinc and gallium phthalocyanine complexes (1 and 2) were synthesized and their inhibition potential of hCA I and II isoenzymes and paraoxonase enzyme were investigated to give direction for further studies. Also, metal effects on carbonic anhydrase and paraoxonase inhibition activity were examined. To the best of our knowledge, even though there are some studies in the literature in which phthalocyanines are evaluated as carbonic anhydrase and α‐glucosidase enzyme inhibitors [19–22], carbonic anhydrase and paraoxonase inhibition activity of anionic water-soluble phthalocyanines has not been reported. Consequently, this is the first study including the evaluation of anionic water-soluble phthalocyanines bearing Ga and Zn metals and the sulfonic acid group as both potential carbonic anhydrase and paraoxonase enzyme inhibitors. Furthermore, molecular docking studies were also applied for a better understanding of the structural and binding profiles of synthesized complexes at the active sites of target enzymes.

## 2. Experimental design

Details on equipment, materials, enzyme inhibition, and molecular docking parameters are supplied as Supporting Information. Water-soluble zinc and gallium phthalocyanine complexes (1 and 2) were prepared according to the reported procedure [14,23]. Biological activity assays and IC50 graphs are provided in Supporting Information.

## 3. Results and discussion

**Figure 1:**
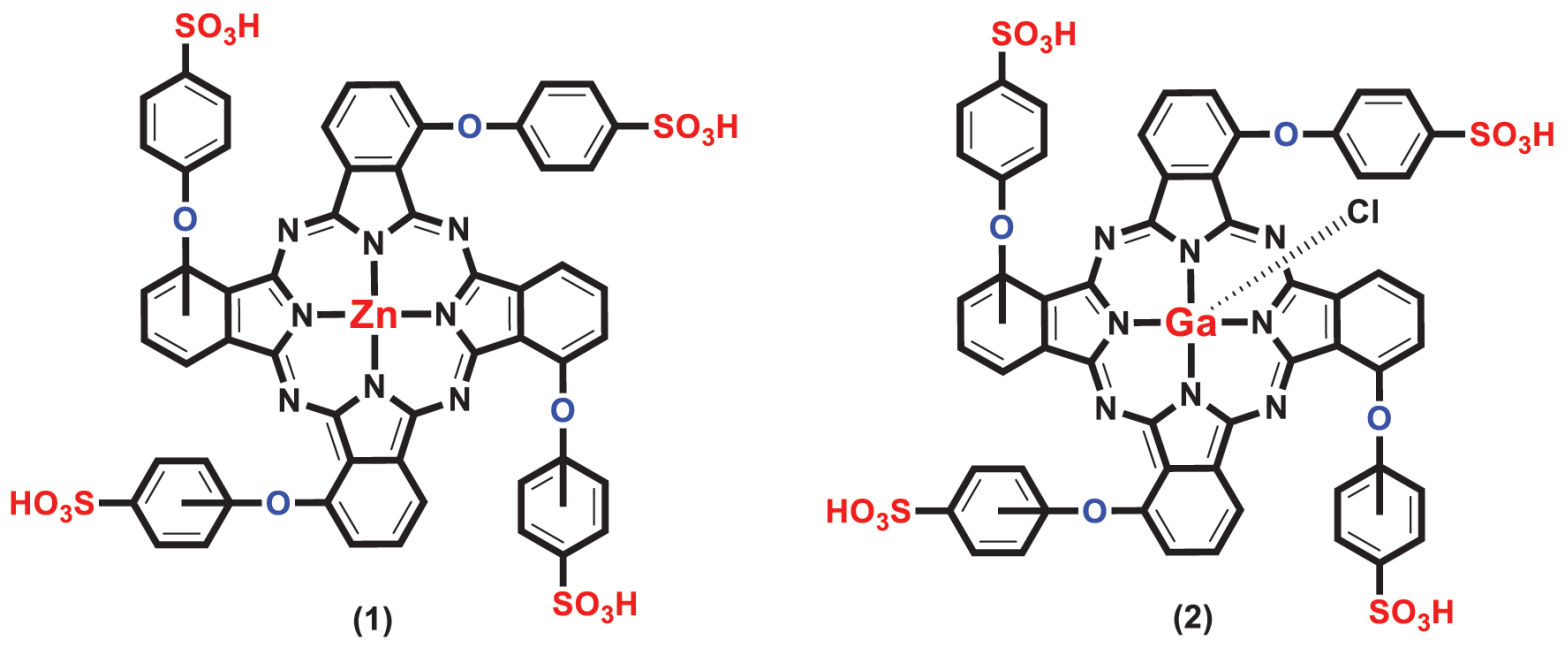
Molecular structure of water-soluble sulfonated zinc and gallium phthalocyanine complexes.

Figure 1 shows the molecular structure of nonperipherally substituted sulfonated zinc and gallium phthalocyanine complexes (1 and 2).

**Figure 2:**
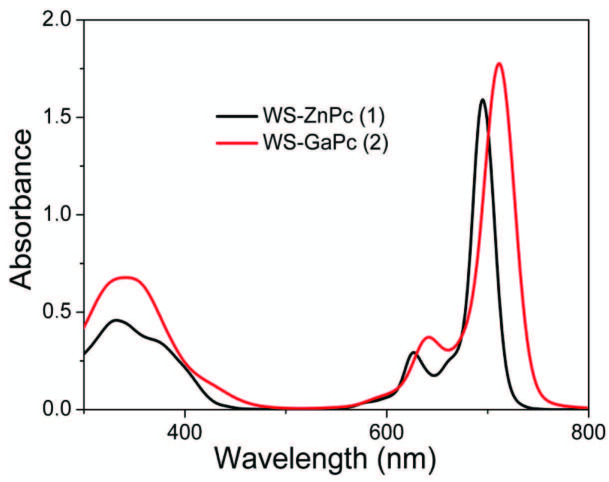
Absorption spectra of water-soluble zinc and gallium phthalocyanine complexes (1 and 2) in DMSO (~ 10 × 10–6 mol dm–3).

The solution spectra show a spectral characteristic indicating D4h symmetry, which is typical for Pc complexes [24]. The absorption peak in the near-UV region is the B-band or Soret band that is attributed to the a2u→e*g transition; a further band in the visible region is related to the Q-band caused by the p–p* transition a1u→e*g. It can be stated that there are some vibrational bands at relatively shorter wavelengths that are a standard property of metallophthalocyanines [25]. The UV-vis absorption spectra of nonperipherally substituted phthalocyanine complexes in DMSO shows two main peaks, the characteristic ligand centered π–π* transitions of a monomeric zinc and gallium phthalocyanine derivatives (1 and 2) with Q-band maxima at 695 and 711 nm, respectively (Figure 2) [14,23,26].

**Figure 3:**
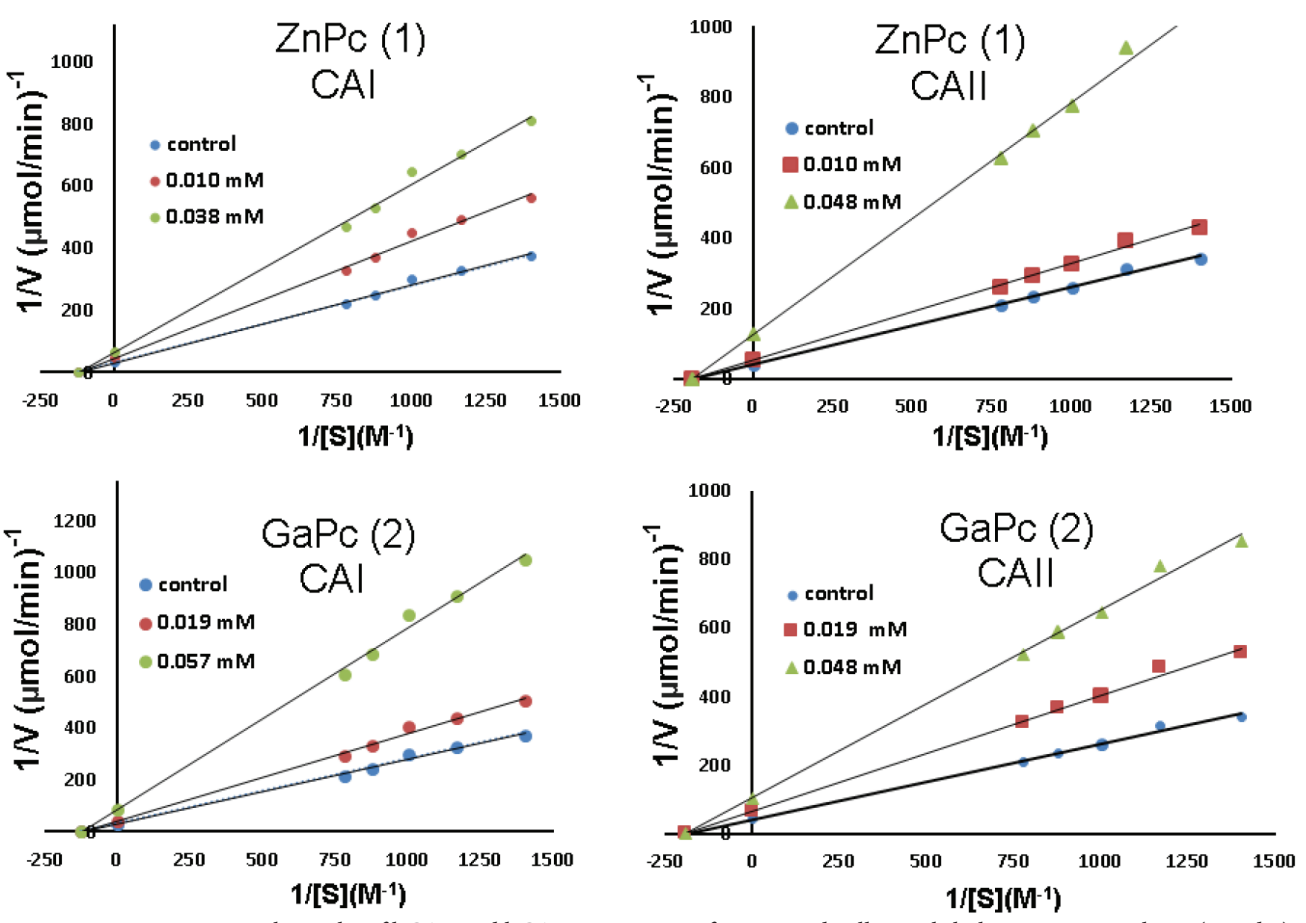
Lineweaver–Burk graphs of hCA I and hCA II isoenzymes for zinc and gallium phthalocyanine complexes (1 and 2).

The CA I and CA II inhibitory activities of the synthesized complexes were determined by hydratase activity (used carbon dioxide as a substrate) and esterase activity (used 4-nitrophenyl-acetate [NPA] as a substrate) assays to calculate the inhibition constants (Ki). Ki values were calculated from the Lineweaver–Burk graphs (Figure 3). The Ki values of the synthesized compounds 1–2 against PON1, hCA I, and hCA II isoforms are given in Table 1. Complexes 1 and 2 inhibited the cytosolic isoforms hCA I and hCA II in the micromolar range (Ki values of 26.09 µM and 43.11 µM for hCA I and 30.95 µM and 33.19 µM for hCA II, respectively). Complex 1, which included Zn metal, had higher inhibitory activity against both hCA I and hCA II with the Ki of 26.09 µM and 30.95 µM, respectively than complex 2 containing Ga metal (Ki values of 43.11 µM against hCA I and 33.19 µM against hCA II). According to these results, it could be considered that the atomic diameter of the Zn metal is larger than Ga; thus, the Zn complex is bulkier than the Ga complex. Their different steric effects could change the inhibitory activity because of an entrance of the active site cavity and the van der Waals interactions with amino acid residues.

**Table 1:** Ki values of complex 1 and 2 against hCA I, hCA II and PON1.

Comp.	Ki (µM) for hCA I	Inhibition type	Ki (µM) for hCA II	Inhibition type	Ki (µM) for PON1	Inhibition type
1	26.09	Noncompetitive	30.95	Noncompetitive	0.37	Noncompetitive
2	43.11	Noncompetitive	33.19	Noncompetitive	0.27	Noncompetitive

Acetazolamide (AAZ) is one of the best-known CAIs and is also used as a standard in CA assays [27]. Whereas complexes 1 and 2 showed much weaker inhibitory activity against hCA I and hCA II than AAZ (Ki of 250 nM and 12.1 nM against hCA I and hCA II, respectively) [28,29], they exhibited higher hCA I and II inhibitory activity than some synthesized compounds (Ki or IC50 values ranging between 75 µM and 620 µM against hCA I, between 126 µM and 427 µM against hCA II) reported as CAIs in the literature [30–33].

The sulphonamides (known as strong CAIs) bind in the deprotonated form to the catalytically critical Zn (II) ion in the enzyme active site [34,35], also contributing an extensive hydrogen bond and van der Waals interactions with amino acid residues of the enzyme active site, as reported in X-ray crystallographic studies of enzyme-inhibitor complexes [36]. We consider that the –SO3H moieties of the synthesized complexes can interact with Zn (II) ion and form the hydrogen bonds with amino acid residues in the enzyme active sites.

**Figure 4:**
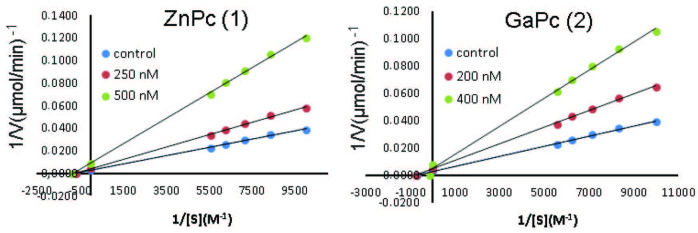
Lineweaver–Burk graphs of paraoxonase1 enzyme for complexes (1 and 2).

On the other hand, the in vitro inhibition effects of synthesized complexes on paraoxonase 1 (PON1) were investigated using paraoxon as a substrate. Both complex 1 and 2 inhibited PON1 (associated with HDL) with Ki of 0.37 µM and 0.27 µM, respectively, as noncompetitive inhibitors (Figure 4). These results demonstrated that complex 1 and 2 have a much stronger PON1 inhibitory activity than the reported PON1 inhibitors (Ki or IC50 values ranging between 35 µM and 550 µM) in the literature [37–39]. Besides, the changing metals and their atomic diameters have not shown a significant effect on PON1 inhibition.

### 3.1. Molecular docking studies

Docking calculations were carried out using the HEX 8.0.0 program [40]. This docking simulation program has allowed the calculation of drug candidate molecules for high atomic weight with a metal center. Docking calculation parameters are correlation type, compute device, FFT mode, and sampling method. These parameters are shape only, CPU, 3D, and range angles, respectively. Grid parameters are solution-100 and step size-(5.5, 5.5, 2.8). Docking simulations for investigated complexes were implemented against 4WR7, 5AML, and 1V04 target proteins representing hCA I, hCA II, and PON1 enzymes, respectively. 4WR7 is the crystal structure of human carbonic anhydrase isozyme I with 2,3,5,6-tetrafluoro-4-(propylthio) benzenesulfonamide. CA is an enzyme that catalyzes reversible carbon dioxide hydration. It is a metalloenzyme that ensures the regulation of acid-base balance and ion transport in all tissues and organs. It is known that increased levels of different CAs are associated with various diseases such as epilepsy and cancer. Since the enthalpy and entropy distribution range of this protein is quite wide, it has a greater entropy contribution to the binding affinity during simulation than enthalpy [41]. 5AML is the three-dimensional structure of human carbonic anhydrase II in complex with 2-(but-2-yn-1-ylsulfamoyl)-4-sulfamoylbenzoic acid. The 5AML target protein has selectivity corresponding to some CA isoforms with medical applications. This protein has a high potential modulus as it contains aliphatic, alkenyl, aralkyl groups, and saccharin derivative substituents [42].

**Figure 5:**
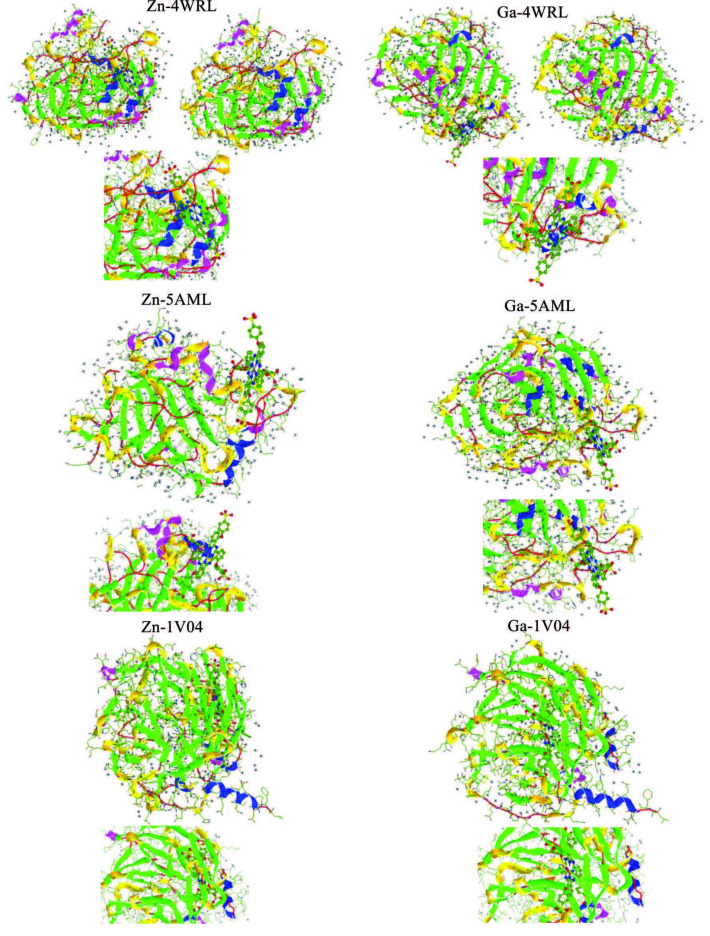
The binding modes between investigated phthalocyanine metal complexes and the determined target proteins.

PON1 is a glycoprotein Ca2+-dependent ester hydrolase, which is synthesized in the liver and is found in HDL in human serum, consisting of 355 amino acids [43]. PON1 is an enzyme with paraoxonase, arylesterase, and lactonase activities that can react with a wide variety of substrates [44]. The three-dimensional structure of a hybrid mammalian recombinant PON1 variant obtained by directed evolution (rePON1) was recently determined providing the first structural information about this hydrolase family, and this structure was named 1V04 [45]. The biological activity of synthesized zinc and gallium phthalocyanine complexes (1) and (2) against hCA I, hCA II, and PON enzymes was attempted to be elucidated by molecular simulation method. Secondary chemical interactions between the amino acid residues of the 4WR7, 5AML, and 1V04 target proteins and the complexes studied were also investigated. In the coupling studies, the estimated free energy of the binding values and the binding modes of the target proteins and metal complexes is given in Table 2 and Figure 5, respectively. According to the docking results in Table 2, the inhibition activities between the complexes and the target proteins, the gallium complex (2) is greater than the inhibition activity of the zinc complex (1). Gallium complex is located in the symmetry cavity of the 4WR7 target protein. Since the gallium complex contains chlorine atoms, it contains a halogen bond in the secondary chemical interaction type. The halogen bond occurs between the chlorine atom bound to the gallium metal and the Gln92 amino acid residue. Also, the gallium complex is in polar interaction with the target protein.

**Table 2:** The docking results between complexes (1) and (2) and the target proteins.

4WR7(hCA I)	(1)	(2)
Binding energy (kcal/mol)	–4.20	–4.88
Type of interaction	H-bond	H-bondHalogenPolar
Binding site	His119	His94, Thr199Gln92Leu198
5AML(hCA II)	(1)	(2)
Binding energy (kcal/mol)	–4.07	–4.13
Type of interaction	H-bond	H-bondHalogen
Binding site	His94	His94, Thr199His200, Asn62
1V04(PON1)	(1)	(2)
Binding Energy (kcal/mol)	–5.25	–5.94
Type of interaction	H-bondPolarpi-pi	H-bondHalogenPolarpi-pi
Binding site	ASP54GLU53, ASP54, ASN227HIS115, TYR236	ASP269HIS115, LEU240, LEU267, ILE291HIS115, ASN168, ASP183, ASN224HIS115, HIS285, PHE292

The increase in the number of secondary chemical interactions between this complex and target proteins may have increased the estimated binding energy. It is clear from the results that in the gallium phthalocyanine complex there are two H-bonds between the His94 and Thr199 amino acid residues of the 4WR7 target protein. This is seen as an H-bond in the zinc complex. The H-bond appears between the zinc phthalocyanine complex and the His199 amino acid residue of the 4WR7 target protein. Both metallophthalocyanine complexes show a similar trend with the 5AML target protein and the 4WR7 target protein. However, in contrast to the interaction of complexes with 5AML target protein, polar interaction is unlike any other.

Zinc and gallium complexes are in a stronger interaction with the 1V04 target protein, which represents the PON1 enzyme, compared to other enzymes. The types of secondary chemical interactions of the compounds with the 1V04 target protein are greater, as seen in Table 2. In the gallium metal-centered complex, unlike in the zinc complex, the halogen bond with the HIS115, LEU240, LEU267, and ILE291 amino acid residues draws attention. The binding energies obtained from the docking results show that the calculated energy values are in a trend parallel with enzyme activities.

As a result, simulation results tend to be similar to experimental inhibition activity. Docking studies are thought to be very important for understanding the chemical interaction mechanism in the inhibition effect.

## 4. Conclusion

In this paper, the investigation of carbonic anhydrase and paraoxonase enzyme inhibition properties of water-soluble sulfonated zinc and gallium phthalocyanines are reported for the first time. The results showed that complex 1 and 2 inhibited the cytosolic isoforms hCA I and hCA II (off-target cytosolic isoforms) in the micromolar range (Ki values of 26.09 µM and 43.11 µM for hCA I and 30.95 µM and 33.19 µM for hCA II, respectively). Moreover, they inhibited PON1 (associated with HDL) with Ki of 0.37 µM and 0.27 µM, respectively. The inhibition kinetics was analyzed by Lineweaver–Burk double reciprocal plots. The analysis revealed that complex 1 and 2 were noncompetitive inhibitors against PON1, hCA I, and hCA II. Whereas complex 1 and 2 showed moderate hCA I and hCA II inhibitory activity, they exhibited strong PON1 inhibitory activity. Furthermore, the changing metals (Zn and Ga) and their atomic diameters affected the CA inhibitory activity, while they did not show a significant effect on PON1. These complexes can be more preferable than other synthetic CA and PON inhibitors due to their high water solubility. Finally, the inhibition efficacy between zinc and gallium complexes and hCA I and hCA II enzymes has been studied in detail with molecular simulation, and experimental data and docking results are highly compatible.
